# The aggregate index of systemic inflammation is positively correlated with the risk of all-cause mortality in sepsis-associated acute kidney injury

**DOI:** 10.1038/s41598-025-16081-7

**Published:** 2025-08-15

**Authors:** Ruming Liu, Yanqi Fan, Bokang Jia, Panshi Li

**Affiliations:** 1 Intensive Care Unit, Dongguan People’s Hospital, The Tenth Affiliated Hospital of Southern Medical University, Dongguan, 523000 Guangdong Province China; 2 Intensive Care Unit, Dongguan People’s Hospital, No.78, Wandao Road, Xingu Yong, Wanjiang Street, Dongguan, Guangdong Province China

**Keywords:** Aggregate index of systemic inflammation (AISI), Sepsis, Sepsis-associated acute kidney injury (SA-AKI), Medical research, Nephrology, Kidney diseases

## Abstract

Sepsis is a major health problem worldwide, and sepsis-associated acute kidney injury (SA-AKI) patients usually experience severe conditions, high mortality, and long length of stay. The predictive value of aggregate index of systemic inflammation (AISI) in the prognosis of several diseases has been documented. This study intends to investigate the association between AISI and mortality in SA-AKI. Data of patients with SA-AKI first admitted to the intensive care unit in 2008–2019 were acquired from the Medical Information Mart for Intensive Care IV (MIMIC-IV). The impact of AISI on 30-/90-/180-d and 1-year mortality in SA-AKI was investigated by Cox proportional hazard regression models, Kaplan-Meier analyses, and restricted cubic spline (RCS) analyses. Moreover, subgroup analyses, stratified by gender, comorbidity, and intervention, were conducted. Totally 9714 SA-AKI patients were included, and they were assigned into a Low AISI Group (AISI < 735.405 × 10^18^/L) and a High AISI Group (AISI ≥ 735.405 × 10^18^/L) based on the median of AISI. As revealed by the regression model, 30-/90-/180-d and 1-year mortality in SA-AKI was higher in the High AISI Group than in the Low AISI Group (*P* < 0.05). Kaplan-Meier analyses confirmed higher 30-/90-/180-d and 1-year survival rates in the Low AISI Group (P_log−rank_<0.0001). Using RCS curves, we also found a nonlinear relation between AISI and 30-/90-/180-d, and 1-year mortality in SA-AKI (P_nonlinear_<0.001). Subgroup analyses suggested no interaction of AISI with the stratified variables (P_interaction_>0.05), and the association of AISI with 30-d mortality was consistent across subgroups. In Conclusion, AISI has an association with mortality in SA-AKI. Quantitative stratification of AISI at admission may contribute to early detection and treatment of SA-AKI with a poor prognosis.

## Introduction

Sepsis is a major health problem with high mortality worldwide^1^and it is a common cause of acute kidney injury (AKI). Sepsis-associated AKI (SA-AKI) affects 60% of sepsis cases^2^. SA-AKI frequently occurs within the first 24 h of admission to intensive care unit (ICU), and it usually corresponds to more severe conditions, higher burden of disease and mortality, greater acute physiologic disorders, and longer length of stay than non-SA-AKI^3^. Due to the high morbidity and mortality of SA-AKI, relevant in-depth research is urgently required, and various prognostic factors should be fully explored and undergo prompt intervention, thereby lowering the mortality in SA-AKI. Therefore, it is of great significance to search for markers of early prediction of SA-AKI prognosis for risk stratification of SA-AKI.

SA-AKI has a high prevalence and involves diverse pathophysiological mechanisms including complement activation, systemic and renal inflammation, mitochondrial dysfunction, renin-angiotensin-aldosterone system disorders, metabolic reprogramming, microcirculatory disorders, and macrocirculatory dysfunction^4^. Since neutrophils, monocytes, platelets, and lymphocytes are important players in the SA-AKI development and progression, some peripheral blood inflammation indicators (e.g. monocyte-to-lymphocyte ratio [MLR], neutrophil-to-lymphocyte platelet ratio [N/LP], and platelet-to-lymphocyte ratio [PLR]) are closely linked to SA-AKI, which have been applied to early warning and diagnosis of SA-AKI^5^. However, rare studies are available on the combination of neutrophil-to-platelet ratio (NPR), neutrophil-to-lymphocyte ratio (NLR), PLR, neutrophil-to-monocyte ratio (NMR), N/LP, NMLR, and MLR for prognostic evaluation of SA-AKI, so further exploration is required. In addition, patient age, gender, comorbidities, severity, baseline health condition, and interventions are also influencing factors for SA-AKI prognosis, so multiple factors should be taken into account in the prognostic evaluation. Aggregate index of systemic inflammation (AISI) (monocyte × neutrophil × platelet/lymphocyte) is superior to NLR, NPR, NMR, NMLR, PLR, N/LP, and MLR in displaying the full picture of blood cells and systemic inflammation status, and it is also an economical and rapidly available indicator. The predictive value of AISI in the prognosis of idiopathic pulmonary fibrosis^6^COVID-19 ^7–10^, hypertension^11^acute myocardial infarction^12^stroke^13,14^peritoneal dialysis^15^and tumors^16–19^ has been documented. However, the association of AISI with SA-AKI prognosis remains inconclusive and has been rarely studied.

This study intends to explore the impact of AISI on SA-AKI mortality and its significance for risk stratification of SA-AKI by using the Medical Information Mart for Intensive Care IV (MIMIC-IV), offering an evidence-based basis for individualized treatment and decision-making and ameliorating the SA-AKI outcomes.

## Methods

### Data source

In this retrospective cohort study, data were acquired from the MIMIC-IV (v2.2), a publicly accessible database containing de-identified data of over 50,000 critically ill cases at Beth Israel Deaconess Medical Center (BIDMC) in 2008–2019, and known for a wealth of clinical data on ICU patients. The Institutional Review Boards of BIDMC and Massachusetts Institute of Technology approved the database use. To meet ethical standards and protect patient privacy, all data used were authenticated and all precautions were taken. The author (Ruming Liu) completed the Protecting Human Research Participants, an NIH web-based course (Record ID: 62088444) and was therefore granted access to the MIMIC-IV. Since de-identified data were utilized, the Ethics Committee of Beth Israel Deaconess Medical Center (BIDMC) waived the consent requirement. This manuscript conforms to the Strengthening the Reporting of Observational Studies in Epidemiology (STROBE) reporting guidelines for observational studies^20^.

## Study population

**Fig. 1:**
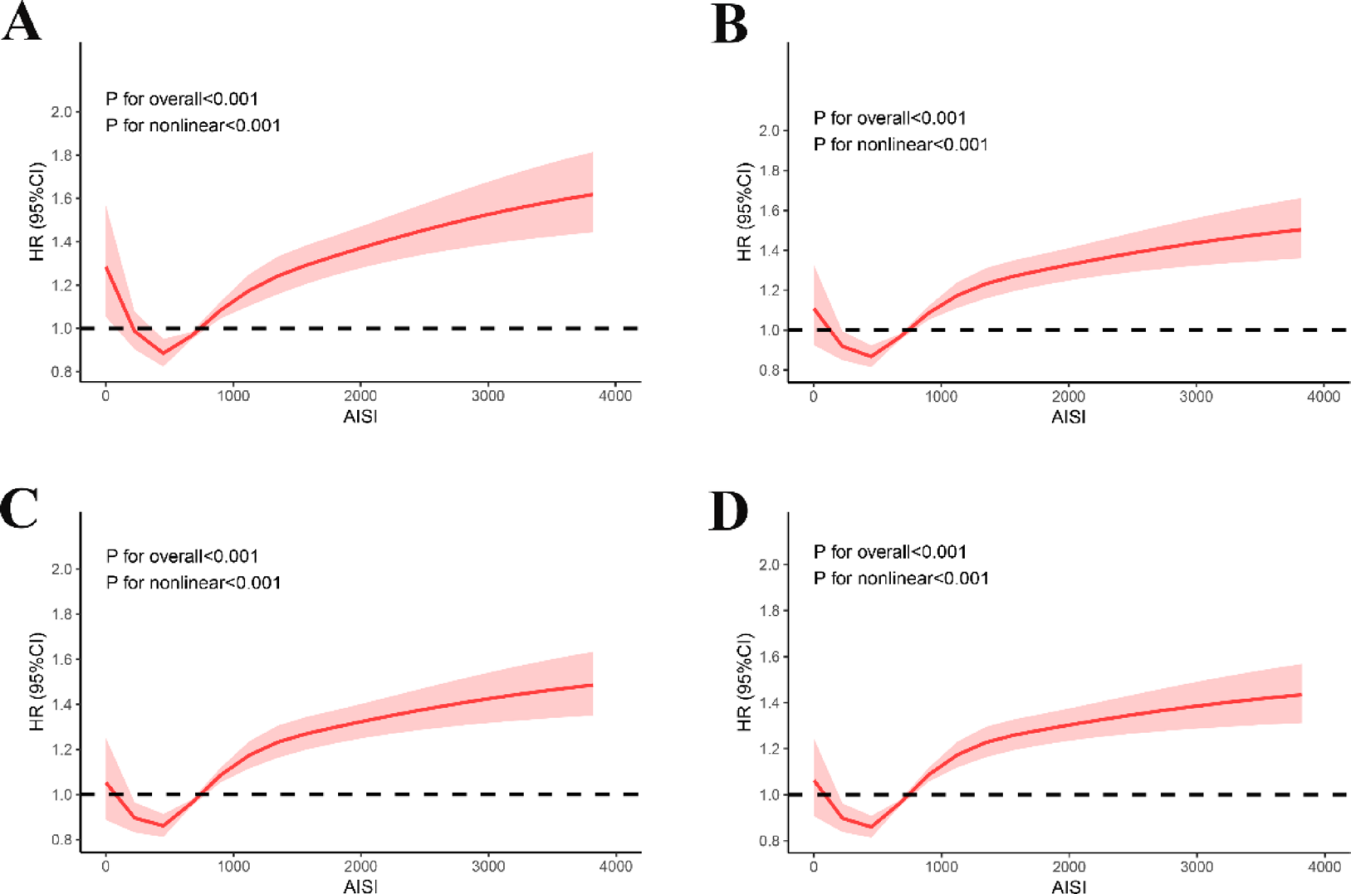
Flow chart showing patient screening and inclusion.

SA-AKI patients who were first admitted to the ICU were selected. Sepsis, as defined by Sepsis-3.0, refers to fatal organ impairment resulting from dysregulation of host responses to infections (SOFA ≥ 2)^21^. A diagnosis AKI is made in the case of an increase in serum creatinine by 1.5-fold from baseline within 7 d or ≥ 0.3 mg/dL (≥ 26.5 µmol/L) within 48 h, or urine volume < 0.5 mL/kg/h for 6 h^22^. SA-AKI refers to the presence of AKI within 7 d of sepsis diagnosis^4^. The study population was all admitted to the ICU and met the diagnostic criteria for sepsis/AKI/SA-AKI. Exclusion criteria: (1) the duration of follow-up < 1 d; (2) missing values in the lymphocyte/monocyte/platelet/neutrophil count; (3) outliers were recorded. For multiple admissions, only the first admission data were recorded. Finally, 9714 patients were included (Fig. 1).

## Calculation of AISI

AISI was calculated as follows: [neutrophil count (10^9^/L) × platelet count (10^9^/L) × monocyte count (10^9^/L)/lymphocyte count (10^9^/L)]^23^.

## Data extraction

The following data (within the first 24 h of ICU admission) were acquired from the MIMIC-IV by SQL: (1) baseline data: gender, race, age, and body mass index; (2) vital signs: temperature_mean, heart_rate_mean, resp_rate_mean, systolic pressure, diastolic pressure, MBP_mean, and SpO_2__mean; (3) laboratory tests: lymphocyte, monocyte, platelet, neutrophil, bilirubin_total, serum creatinine, alanine aminotransferase, aspartate aminotransferase, glucose_mean, lactate, prothrombin time, and partial thromboplastin time; (4) scores: Glasgow Coma Scale, and Simplified Acute Physiology Score II; (5) comorbidities: type 2 diabetes (T2D), coronary atherosclerotic heart disease, chronic obstructive pulmonary disease, cirrhosis, congestive heart failure, cerebrovascular disease, and peptic ulcer; (6) interventions: antibiotic, norepinephrine, ventilation_status, and continuous renal replacement therapy. For laboratory indicators measured multiple times within 24 h of ICU admission, the percentage of missing values was calculated for each continuous variable to minimize bias caused by sample exclusion. For variables with a percentage of missing values < 30%, the missing values were predicted by random forest-based multiple imputation, and the results of five predictions were averaged. We excluded the variables with too many missing values (> 30%).

## Outcomes

The primary outcome was 30-d mortality, and the secondary outcomes were 90-d, 180-d, and 1-year mortality.

### Statistical analysis

Continuous variables were presented by mean ± standard deviation or median (interquartile range) as appropriate, and underwent a t-test or a nonparametric test. Categorical variables were described by frequencies and percentages, and underwent a chi-square test. To identify the association of AISI with mortality, we utilized Cox proportional hazard regression models to estimate the hazard ratios (HRs) with 95% confidence intervals (CIs). The patients were assigned into a Low AISI Group (AISI < 735.405 × 10^18^/L) and a High AISI Group (AISI ≥ 735.405 × 10^18^/L) based on the median of AISI. To enhance the robustness of results, the regression estimates were corrected by Model 1 (unadjusted), Model 2 (adjusted for gender, age, and race), and Model 3 (adjusted for all covariates). Moreover, the potential nonlinear relation between AISI and mortality was presented by restricted cubic spline (RCS) analyses. Kaplan-Meier survival curves were plotted to compare the survival rate between the two groups (log-rank test). Furthermore, subgroup and interaction analyses were conducted on the primary outcome, and the former was stratified by gender (male/female), comorbidities (COPD, CAHD, cerebrovascular disease, cirrhosis, congestive heart failure, peptic ulcer disease, and T2D), norepinephrine, antibiotic, ventilation status, and Crrt mode. R4.4.1 was utilized, and *P* < 0.05 (two-sided) suggested statistical significance.

## Results

### Baseline characteristics

According to the eligibility criteria, 9714 SA-AKI patients were finally included, of which 5629 (57.9%) were males and 4085 (42.1%) were females. They consisted of 7687 (79.1%) 30-d survivors and 2027 (20.9%) 30-d deaths. The High AISI Group primarily included patients with higher age, mean heart rate, mean temperature, mean blood glucose, mean respiratory rate, neutrophil count, monocyte count, platelet count, SAPS II score, AST, and PT, non-CRRT, non-cirrhosis, non-COPD, and non-peptic ulcer patients, and antibiotic-treated patients (*P* < 0.05) (Table 1).

**Table 1:** Baseline characteristics.

Variables	level	Overall	AISI (10^18^/L)		*P* value
			< 735.405	≥ 735.405	
N		9714	4857	4857	
Gender (%)	Female	4085 (42.1)	2019 (41.6)	2066 (42.5)	0.344
	Male	5629 (57.9)	2838 (58.4)	2791 (57.5)	
Age (years)		68.21 [57.52, 78.40]	67.29 [56.91, 77.40]	69.29 [58.35, 79.51]	< 0.001
Race (%)	ASIAN	169 (1.7)	104 (2.1)	65 (1.3)	< 0.001
	BLACK	1038 (10.7)	640 (13.2)	398 (8.2)	
	Other	2153 (22.2)	984 (20.3)	1169 (24.1)	
	WHITE	6354 (65.4)	3129 (64.4)	3225 (66.4)	
BMI (kg/m^2^)		28.30 [24.30, 33.30]	28.30 [24.50, 33.00]	28.20 [24.10, 33.70]	0.322
Vital signs					
Temperature mean (°C)		36.85 [36.64, 37.14]	36.84 [36.62, 37.12]	36.86 [36.65, 37.16]	< 0.001
Heart rate mean (bpm)		84.61 [74.95, 97.04]	83.42 [74.25, 95.58]	85.92 [75.93, 98.21]	< 0.001
Resp rate mean (bpm)		19.22 [17.00, 22.08]	18.78 [16.71, 21.69]	19.60 [17.26, 22.43]	< 0.001
SP (mmHg)		128.00 [116.00, 140.00]	128.00 [116.00, 140.00]	128.00 [116.00, 140.00]	0.546
DP (mmHg)		72.00 [64.00, 80.00]	73.00 [64.00, 80.00]	72.00 [64.00, 80.00]	0.195
mBP mean (mmHg)		75.93 [70.41, 83.34]	75.94 [70.48, 83.24]	75.93 [70.29, 83.48]	0.906
SPO_2_ mean (%)		97.00 [95.56, 98.38]	97.10 [95.71, 98.43]	96.88 [95.39, 98.31]	< 0.001
Laboratory parameters					
Neutrophil (10^9^/L)		6.94 [4.47, 10.94]	4.63 [3.38, 6.37]	10.35 [7.52, 14.56]	< 0.001
Lymphocyte (10^9^/L)		1.24 [0.80, 1.85]	1.50 [1.00, 2.13]	1.04 [0.66, 1.52]	< 0.001
Monocyte (10^9^/L)		0.68 [0.47, 0.96]	0.54 [0.38, 0.70]	0.89 [0.66, 1.20]	< 0.001
Platelet (10^9^/L)		216.00 [164.00, 276.00]	190.00 [141.00, 244.00]	242.00 [189.00, 308.00]	< 0.001
ALT (U/L)		24.00 [16.00, 42.00]	24.00 [16.00, 40.00]	24.00 [16.00, 45.00]	0.105
AST (U/L)		29.00 [20.00, 56.00]	28.00 [20.00, 53.00]	29.00 [20.00, 59.00]	0.017
Bilirubin total (mg/dL)		0.50 [0.30, 1.00]	0.50 [0.30, 1.00]	0.50 [0.30, 0.90]	0.539
Creatinine (mg/dL)		1.00 [0.80, 1.40]	1.00 [0.80, 1.30]	1.00 [0.80, 1.50]	< 0.001
Glucose mean (mg/dL)		132.00 [112.62, 163.72]	131.00 [113.00, 160.83]	133.20 [112.00, 167.00]	0.023
Lactate (mmol/L)		1.70 [1.20, 2.60]	1.70 [1.20, 2.50]	1.70 [1.20, 2.70]	< 0.001
PT (s)		12.70 [11.50, 14.80]	12.50 [11.40, 14.50]	12.90 [11.70, 15.10]	< 0.001
PTT (s)		29.80 [26.60, 34.30]	30.20 [27.00, 34.90]	29.40 [26.30, 33.60]	< 0.001
Scoring					
GCS (points)		14.00 [13.00, 15.00]	14.00 [13.00, 15.00]	14.00 [13.00, 15.00]	< 0.001
SAPS II (median points)		38.00 [30.00, 47.00]	37.00 [29.00, 46.00]	39.00 [31.00, 48.00]	< 0.001
Comorbidities (%)					
Type 2 diabetes (%)	No	6464 (66.5)	3206 (66.0)	3258 (67.1)	0.273
	Yes	3250 (33.5)	1651 (34.0)	1599 (32.9)	
CAHD (%)	No	5731 (59.0)	2843 (58.5)	2888 (59.5)	0.364
	Yes	3983 (41.0)	2014 (41.5)	1969 (40.5)	
COPD (%)	No	7859 (80.9)	4028 (82.9)	3831 (78.9)	< 0.001
	Yes	1855 (19.1)	829 (17.1)	1026 (21.1)	
Cirrhosis (%)	No	8436 (86.8)	4017 (82.7)	4419 (91.0)	< 0.001
	Yes	1278 (13.2)	840 (17.3)	438 (9.0)	
Congestive heart failure (%)	No	8236 (84.8)	4095 (84.3)	4141 (85.3)	0.204
	Yes	1478 (15.2)	762 (15.7)	716 (14.7)	
Cerebrovascular disease (%)	No	8691 (89.5)	4330 (89.1)	4361 (89.8)	0.321
	Yes	1023 (10.5)	527 (10.9)	496 (10.2)	
Peptic ulcer disease (%)	No	9479 (97.6)	4719 (97.2)	4760 (98.0)	0.008
	Yes	235 (2.4)	138 (2.8)	97 (2.0)	
Curing					
Antibiotic (%)	No	368 (3.8)	226 (4.7)	142 (2.9)	< 0.001
	Yes	9346 (96.2)	4631 (95.3)	4715 (97.1)	
Norepinephrine (%)	No	9428 (97.1)	4703 (96.8)	4725 (97.3)	0.208
	Yes	286 (2.9)	154 (3.2)	132 (2.7)	
Ventilation status (%)	No	3560 (36.6)	1771 (36.5)	1789 (36.8)	0.72
	Yes	6154 (63.4)	3086 (63.5)	3068 (63.2)	
CRRT (%)	No	8854 (91.1)	4458 (91.8)	4396 (90.5)	0.029
	Yes	860 (8.9)	399 (8.2)	461 (9.5)	
Status					
Status 30d (%)	survival	7687 (79.1)	3990 (82.1)	3697 (76.1)	< 0.001
	death	2027 (20.9)	867 (17.9)	1160 (23.9)	
Status 90d (%)	survival	7136 (73.5)	3754 (77.3)	3382 (69.6)	< 0.001
	death	2578 (26.5)	1103 (22.7)	1475 (30.4)	
Status 180d (%)	survival	6792 (69.9)	3609 (74.3)	3183 (65.5)	< 0.001
	death	2922 (30.1)	1248 (25.7)	1674 (34.5)	
Status 1year (%)	survival	6362 (65.5)	3401 (70.0)	2961 (61.0)	< 0.001
	death	3352 (34.5)	1456 (30.0)	1896 (39.0)	

### Association of AISI with mortality in SA-AKI

**Fig. 2:**
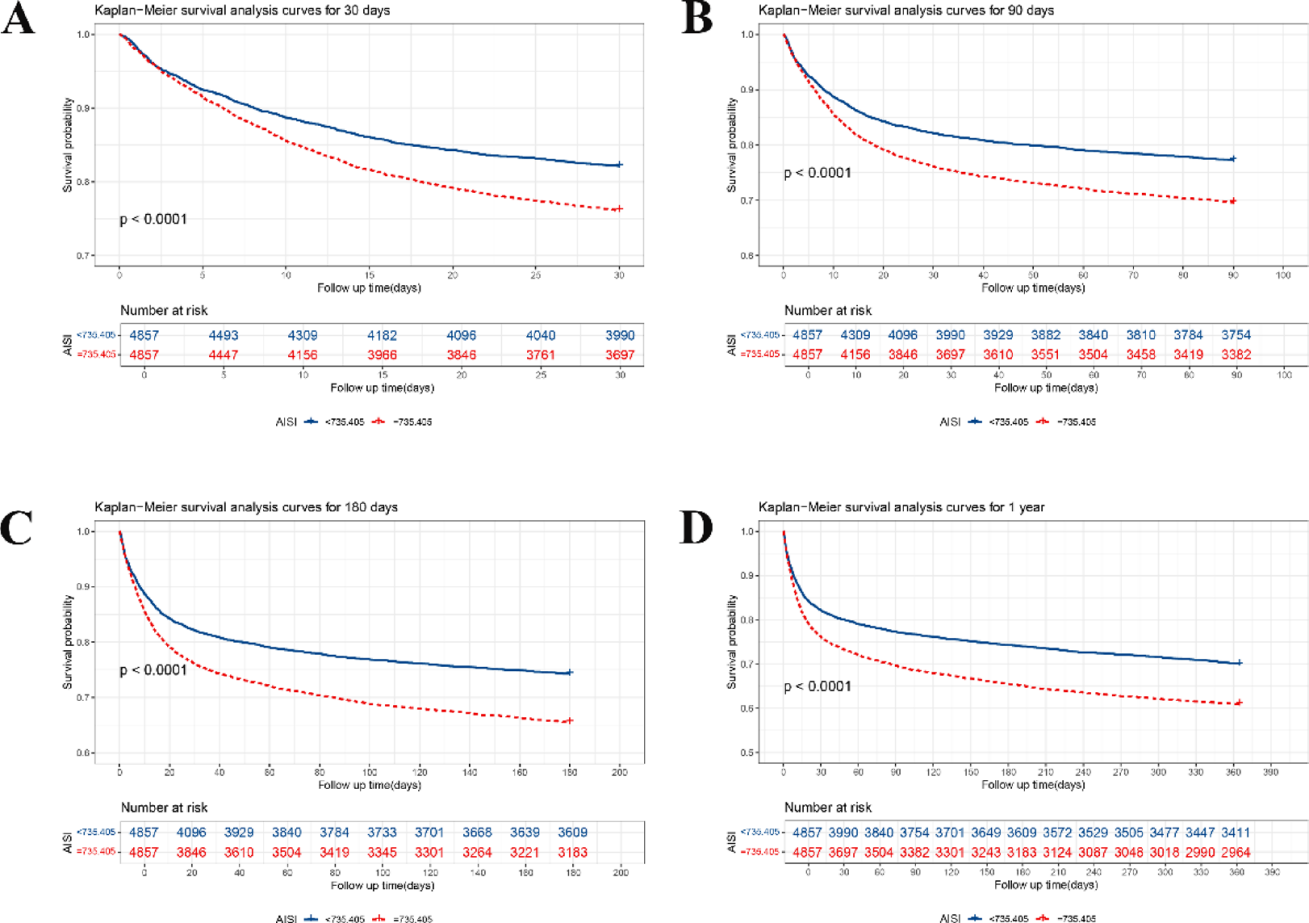
Comparison of Kaplan–Meier survival curves between groups: (**A**) 30-day survival curves; (**B**) 90-day survival curves; (**C**) 180-day survival curves; (**D**) 1-year survival curves.

The association of AISI with mortality in SA-AKI was assessed by HR with 95% CI using Cox proportional hazard regression models (Table 2). We found a higher 30-d mortality in the High AISI Group (*P* < 0.05). Specifically, the 30-d mortality in the High AISI Group (H735.405 × 10^18^/L) was 1.377 [HR (95% CI): 1.377 (1.261–1.504)], 1.313 [HR (95% CI): 1.313 (1.202–1.435)], and 1.184 times [HR (95% CI): 1.184 (1.082–1.296)] that in the Low AISI Group (< 735.405 × 10^18^/L), respectively, in Model 1, Model 2, and Model 3. The High AISI Group also had higher 90-/180-d and 1-year mortality (*P* < 0.05), and Kaplan-Meier analyses confirmed higher 30-/90-/180-d and 1-year survival rates in the Low AISI Group (P_log−rank_<0.0001) (Fig. 2).

**Table 2:** Multivariable Cox regression analysis for mortality.

	AISI (10^18^/L)	Model 1		Model 2		Model 3	
		HR(95%CI)	*P* value	HR(95%CI)	*P* value	HR(95%CI)	*P* value
30d	< 735.405	—	—	—	—	—	—
	≥ 735.405	1.377(1.261–1.504)	< 0.001	1.313(1.202–1.435)	< 0.001	1.184(1.082–1.296)	< 0.001
90d	< 735.405	—	—	—	—	—	—
	≥ 735.405	1.395(1.290–1.508)	< 0.001	1.335(1.234–1.444)	< 0.001	1.221(1.127–1.323)	< 0.001
180d	< 735.405	—	—	—	—	—	—
	≥ 735.405	1.411(1.311–1.518)	< 0.001	1.356(1.260–1.460)	< 0.001	1.243(1.153–1.340)	< 0.001
1year	< 735.405	—	—	—	—	—	—
	≥ 735.405	1.385(1.294–1.483)	< 0.001	1.339(1.251–1.435)	< 0.001	1.240(1.156–1.330)	< 0.001

Model 1: unadjusted

Model 2: adjusted for gender, age, and race

Model 3: adjusted for gender, age, race, BMI, temperature mean, heart rate mean, resp rate mean, SP, DP, mBP mean, SPO2 mean, bilirubin total, creatinine, ALT, AST, glucose mean, lactate, PT, PTT, GCS, SAPS II, Type 2 diabetes, CAHD, COPD, cirrhosis, congestive heart failure, cerebrovascular disease, peptic ulcer disease, antibiotic, norepinephrine, ventilation status, and CRRT.

AISI, aggregate index of systemic inflammation; BMI, body mass index; SP, systolic pressure; DP, diastolic pressure; mBP, mean blood pressure; SPO2, percutaneous oxygen saturation; ALT, Alanine aminotransferase; AST, Aspartate aminotransferase; PT, Prothrombin time; PTT, partial thromboplastin time; GCS, Glasgow Coma Scale; SAPS II, simplified acute physiology score II; CRRT, continuous renal replacement therapy; CAHD, coronary atherosclerotic heart disease; COPD, chronic obstructive pulmonary disease; HR, hazard ratio; CI, confidence interval.

### RCS analyses

**Fig. 3:**
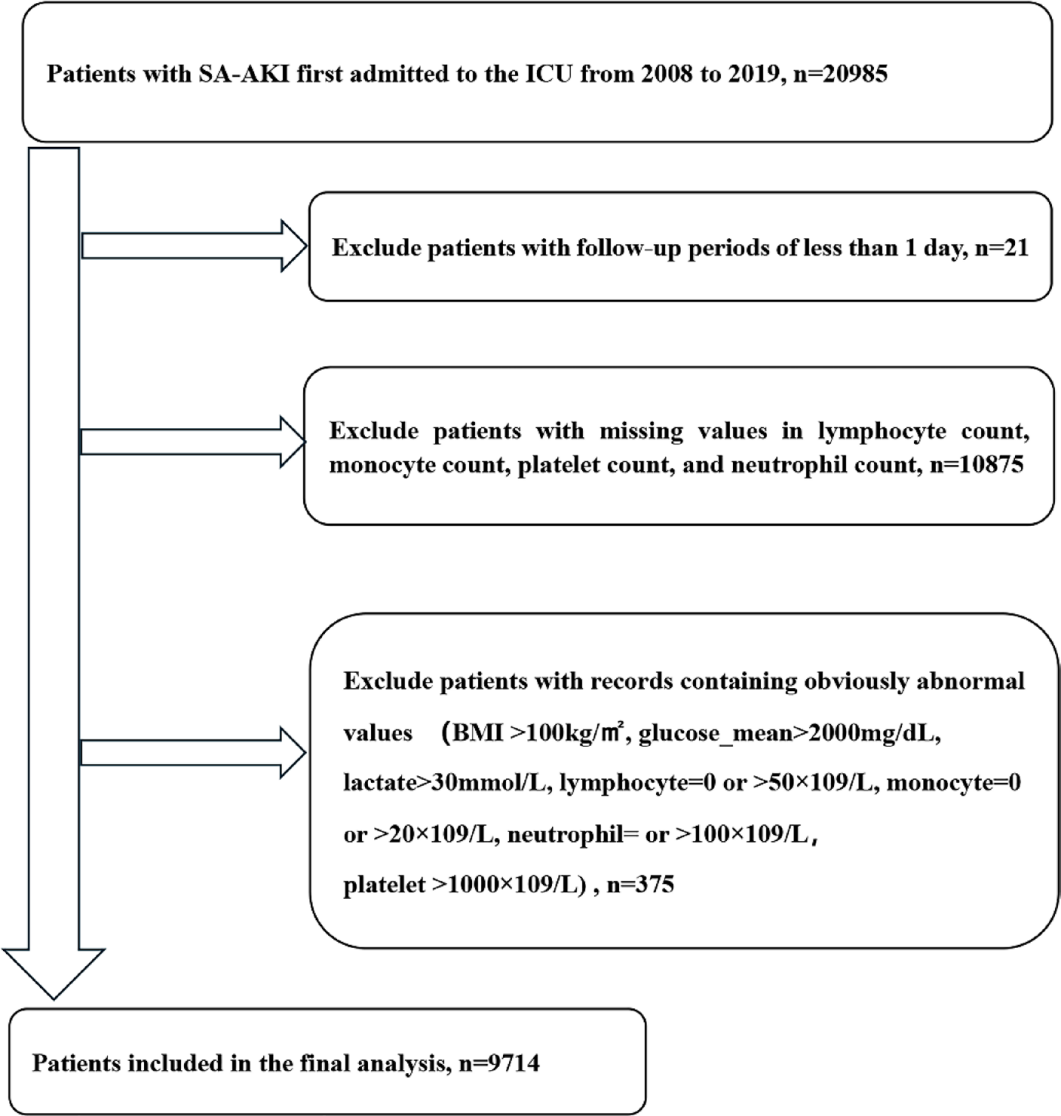
Nonlinear relationship between AISI and all-cause mortality in SA-AKI: (**A**) 30-day mortality; (**B**) 90-day mortality; (**C**) 180-day mortality; (**D**) 1-year mortality.

The association of AISI with mortality in SA-AKI was visualized by RCS curves (Fig. 3), and we found a nonlinear relation between AISI and 30-d mortality in SA-AKI (P_nonlinear_<0.001). In this J-shaped relation, the cutoff of AISI was 450.117 × 10^18^/L. The mortality in SA-AKI rose with the increase in AISI when AISI > 450.117 × 10^18^/L. Similarly, the 90-/180-d and 1-year mortality in SA-AKI displayed nonlinear relations with AISI (P_nonlinear_<0.001). In the threshold analysis, the HR value for 30-d mortality in patients with AISI < 450.117 × 10¹⁸/L (Table 3) was 0.517 (95% CI: 0.424–0.631) (*P* < 0.001), indicating that for each unit increase in AISI, the 30-d mortality decreased by 48.3%. In contrast, AISI and 30-d mortality were positively correlated with each other when AISI ≥ 450.117 × 10¹⁸/L [HR (95% CI): 3.600 (2.740–4.731)] (Table 3). Similar results were observed for 90-d, 180-d, and 1-year mortality.

**Table 3:** The threshold effects of AISI and SA-AKI were analyzed using a two-segment regression model.

AISI	Adjusted HR	95%CI	*P* value
Likelihood ratio test			
30d			
< 450.117	0.517	0.424,0.631	< 0.001
≥ 450.117	3.600	2.740,4.731	< 0.001
90d			
< 450.117	0.569	0.466,0.696	< 0.001
≥ 450.117	3.772	2.958,4.810	< 0.001
180d			
< 450.117	0.603	0.492,0.738	< 0.001
≥ 450.117	3.835	3.051,4.821	< 0.001
1year			
< 450.117	0.619	0.510,0.752	< 0.001
≥ 450.117	3.616	2.919,4.481	< 0.001

### Subgroup analyses

As shown in Table 4, the association of AISI with 30-d mortality in SA-AKI was more significant in the subgroups of male [HR (95% CI): 1.215 (1.077–1.371)], antibiotic [HR (95% CI): 1.180 (1.078–1.293)], mechanical ventilation [HR (95% CI): 1.182 (1.059–1.321)], non-mechanical ventilation [HR (95% CI): 1.206 (1.027–1.415)], non-CRRT [HR (95% CI): 1.185 (1.072–1.310)], non-norepinephrine [HR (95% CI): 1.189 (1.085–1.302)], non-COPD [HR (95% CI): 1.207 (1.091–1.334)], CAHD [HR (95% CI): 1.228 (1.064–1.418)], non-CAHD [HR (95% CI): 1.152 (1.024–1.297)], non-cerebrovascular disease [HR (95% CI): 1.193 (1.085–1.312)], non-cirrhosis [HR (95% CI): 1.196 (1.083–1.322)], congestive heart failure [HR (95% CI): 1.467 (1.125–1.912)], non-congestive heart failure [HR (95% CI): 1.144 (1.039–1.259)], non-peptic ulcer [HR (95% CI): 1.185 (1.082–1.297)], T2D [HR (95% CI): 1.211(1.028–1.427)], and non-T2D [HR (95% CI): 1.157 (1.038–1.291)] (*P* < 0.05). Subgroup analyses suggested no interaction of AISI with the stratified variables (P_interaction_>0.05), and the association of AISI with 30-d mortality was consistent across subgroups. To sum up, the stratified variables had no influence on the association of AISI with 30-d mortality in SA-AKI, and AISI acted as an independent factor influencing 30-d mortality in SA-AKI.

**Table 4:** Subgroup analyses.

	HR(95%CI)	*P* value	*P* for interaction
Gender			0.592
Male	1.215(1.077–1.371)	0.002	
Female	1.138(0.992–1.305)	0.065	
COPD			0.464
Yes	1.052(0.855–1.294)	0.635	
No	1.207(1.091–1.334)	< 0.001	
Antibiotic			0.609
Yes	1.180(1.078–1.293)	< 0.001	
No	1.395(0.645–3.019)	0.398	
CAHD			0.286
Yes	1.228(1.064–1.418)	0.005	
No	1.152(1.024–1.297)	0.019	
Cerebrovascular disease			0.870
Yes	1.139(0.848–1.529)	0.387	
No	1.193(1.085–1.312)	< 0.001	
Cirrhosis			0.280
Yes	1.083(0.865–1.356)	0.488	
No	1.196(1.083–1.322)	< 0.001	
Congestive heart failure			0.094
Yes	1.467(1.125–1.912)	0.005	
No	1.144(1.039–1.259)	0.006	
Peptic ulcer disease			0.668
Yes	1.160(0.448–3.007)	0.76	
No	1.185(1.082–1.297)	< 0.001	
Type 2 Diabetes			0.708
Yes	1.211(1.028–1.427)	0.022	
No	1.157(1.038–1.291)	0.009	
Ventilation status			0.895
Yes	1.182(1.059–1.321)	0.003	
No	1.206(1.027–1.415)	0.022	
CRRT			0.316
Yes	1.177(0.948, 1.460)	0.14	
No	1.185(1.072–1.310)	< 0.001	
Norepinephrine			0.725
Yes	1.323(0.588–2.976)	0.499	
No	1.189(1.085–1.302)	< 0.001	

CRRT, continuous renal replacement therapy; CAHD, coronary atherosclerotic heart disease; COPD, chronic obstructive pulmonary disease; HR, hazard ratio; CI, confidence interval.

### Sensitivity analysis

To guarantee the accuracy of AISI calculation and its association with mortality, we excluded patients with diseases that might affect AISI and fitted three COX regression models. The results revealed that a significant positive correlation was still present between AISI and mortality in SA-AKI patients. The 30-d mortality in the High AISI Group (≥ 735.405 × 10^18^/L) was 1.371 [HR (95% CI): 1.371 (1.251–1.502)], 1.303 [HR (95% CI): 1.303 (1.189–1.429)], and 1.174 times [HR (95% CI): 1.174 (1.069–1.290)] that in the Low AISI Group (< 735.405 × 10^18^/L), respectively, in Model 1, Model 2, and Model 3. Similar results were observed for 90-d, 180-d, and 1-year mortality (Table S1).

## Discussion

SA-AKI has complex pathophysiologic mechanisms involving systemic inflammatory response, microcirculatory disorders, and immune dysregulation. This study revealed a significant correlation of AISI, calculated by neutrophil, monocyte, platelet, and lymphocyte counts, with mortality in SA-AKI, and in this J-shaped relation, the cutoff of AISI was 450.117 × 10^18^/L; higher mortality in the High AISI Group, and a higher survival rate in the Low AISI Group. Subgroup analyses suggested that the association of AISI with 30-d mortality was consistent across subgroups. The findings offer a new perspective on clinical risk stratification.

Inflammation and immune response are known as key factors in sepsis development and progression^24^. Neutrophils, monocytes, platelets, and lymphocytes are important components of the immune system and are strongly implicated in the sepsis onset and development. NLR plus NPR is a reliable predictor of 28-d mortality in sepsis^25^whereas the association of NMR with 28-d mortality in sepsis remains inconclusive and to be further explored by a larger sample size, standardized methods, and specific patient groups. In addition, NMLR also exhibits value as an indicator for sepsis risk assessment and intervention^26^. A U-shaped relation of PLR at admission with mortality in sepsis has also been found, and an early decline in PLR indicates an elevation of in-hospital mortality^27^.

A variety of blood count-derived inflammation indicators are available for prognostic prediction of AKI. For example, PLR is used to predict the AKI prognosis in critically ill cases^28^. Changes in absolute monocyte and lymphocyte counts may reflect the pro-/anti-inflammatory balance in AKI, and MLR is used to predict short-term mortality in AKI in critically ill patients^29^. Moreover, MLR is positively correlated with C-reactive protein and procalcitonin, and initial MLR and NLR can serve as independent risk factors for AKI in critically ill cases, both of which, however, are weak in predicting in-hospital mortality^30^. Elevation of N/LP leads to a higher risk of SA-AKI and severe AKI within 7 d of ICU admission, which is more significant in males, the elderly, infectious shock, and poor health^31^.

AISI is a novel inflammation indicator derived from the complete blood count, which integrates peripheral blood neutrophils, monocytes, lymphocytes, and platelets, and outperforms PLR, MLR, NLR, N/LP, NPR, NMR, and NMLR in indicating the dynamic balance between pro-inflammatory (neutrophils, monocytes, platelets) and anti-inflammatory cells (lymphocytes). Elevation of AISI suggests the presence of both systemic inflammatory over-activation and immunosuppression. The important value of AISI in the prognostic evaluation of various diseases, and its great correlation with mortality in idiopathic pulmonary fibrosis have been documented^6^. Current research on inflammation has revealed a link between inflammatory processes and tumor transformation, progression, metastasis, and relapse^32^. A systematic review and meta-analysis identified AISI as a better predictor of cancer prognosis and higher AISI as a poor prognostic factor for non-metastatic or metastatic diseases and in targeted or immunotherapy^17^. It is reported that AISI is a better predictor of prognosis in late-stage non-small cell lung cancer^16^ and a potential marker for predicting the risk of prostate cancer progression^19^and higher AISI is also linked to a poorer prognosis in esophageal cancer^18^. Besides, inflammation is crucial in the onset and progression of cardiovascular diseases (e.g. atherosclerosis)^33^. Research suggests that AISI may act as an early warning indicator of poor prognosis in acute myocardial infarction^12^and that elevated AISI corresponds to an increased risk of cardiovascular mortality in hypertensive adults^11^. AISI also stays higher in stroke, especially hemorrhagic stroke, and it is related to mortality when beyond a certain limit, which can predict the stroke prognosis^13,14^. In addition, AISI can predict mortality in infectious diseases (e.g. COVID-19)^10^and its predictive value in sepsis mortality has also been verified in another retrospective study^34^. However, the impact of AISI on mortality in SA-AKI is rarely studied.

In this study, mortality greatly increased in the High AISI Group and displayed a J-shaped relation with AISI, which was considered to be possibly related to the synergy of inflammatory storm and immunosuppression. In the case of elevation of AISI, over-activation of neutrophils and monocytes results in an inflammatory storm that directly damages renal tubular epithelial cells and microvascular endothelial cells. Platelets are involved in microthrombosis and worsen local ischemia, and decreases in lymphocytes lead to immunosuppression and proneness to secondary infections. AISI, by dynamically reflecting the inflammatory and immune statuses, allows for early identification of high-risk groups, and its cutoff (450.117 × 10¹⁸/L) lays a quantitative basis for clinical risk stratification and interventions. Subgroup analyses suggested no interaction of AISI with the stratified variables, and AISI was an independent factor influencing 30-d mortality in SA-AKI.

SA-AKI is a prevalent complication of sepsis. As parts of the innate immune system, neutrophils, natural killer cells, and macrophages are implicated in AKI^35^. In sepsis, neutrophils are key players in the host inflammatory response to pathogens, which function mainly by degranulation, phagocytosis, and neutrophil extracellular trap (NET) release; due to over-activation of neutrophils and NET release, endothelial cells will shift from an anticoagulant/anti-inflammatory phenotype to a pro-coagulant/pro-inflammatory phenotype, and NETs also degrade the glycocalyx and enhance endothelial permeability, resulting in endothelial barrier instability and further facilitating sepsis progression^36^. As core effector cells of the early inflammatory response in sepsis, neutrophils are activated and recruited to the kidney in the systemic inflammatory response, which not only act as an indicator and amplifier of the inflammatory response but also cause renal damage by obstructing microvessels and secreting toxic molecules (e.g., proteases, oxides, cytokines, NETs)^37^. Moreover, monocytes and neutrophils jointly initiate the inflammatory response. In the early stage of AKI, monocyte-derived macrophages release chemokines that recruit neutrophils to affected sites^25^worsen renal damage by promoting the inflammatory cell aggregation and the expression of inflammatory mediators, and also secrete exosomes that may be involved in the SA-AKI onset and progression^38^. The severity of sepsis may be associated with a reduction in monocyte counts, low monocyte counts may lead to out-of-control localized infections and affect prognosis, and the initial absolute monocyte count at admission is an independent factor influencing 28-d mortality in severe sepsis^39^. Platelets are also key to the resistance to infection and are implicated in immune promotion and coagulation activation^40^. Platelets interact with neutrophils to participate in the NET formation^41^and interact with antigen-presenting cells and T/B cells to regulate the immune response^42^. In the early stage of sepsis, by interacting with platelet hemostatic receptors (e.g. GPIIb/IIIa, GPIb) and immune receptors (e.g. FcγRIIa, TLRs), pathogens induce platelet activation, aggregation, and consumption, trigger systemic thrombosis, and obstruct the microvascular system, leading to ischemic injury to peripheral tissues and even organ failure. Platelet counts often increase in response to such platelet consumption, and thrombocytopenia occurs when the rate of platelet consumption exceeds that of production^43^. Moreover, platelet activation may play a role in AKI pathology^44^. Lymphocytes, important cells of the adaptive immune system, are involved in sepsis-induced immunosuppression^45^. Lymphopenia is a marker for sepsis-induced immunosuppression prominently characterized by apoptosis-induced early massive loss of lymphocytes and expansion of immunosuppressive cells (IL-10-producing B cells, regulatory T cells, and myeloid-derived suppressor cells). As a result, patients will die due to latent viral reactivation and susceptibility to secondary opportunistic infections^46^. Apoptosis of lymphocytes results in severe and persistent lymphopenia in septic shock, producing a poor prognosis^47^. In addition, lymphocytes also secrete cytokines to regulate inflammatory responses and induce kidney injury^48^. To sum up, the mechanism of AISI as a potential marker of SA-AKI prognosis has not been clarified, but it, integrating neutrophil, monocyte, platelet, and lymphocyte counts, can reflect more comprehensively and objectively the inflammatory changes.

This retrospective cohort study first revealed a J-shaped nonlinear relation of AISI with SA-AKI prognosis. However, some limitations are worth noting. First, the data were collected retrospectively. Second, no information on the site of infection and etiological examination results was reported. Third, only the value of AISI calculated first after admission rather than its changes during hospitalization was used, which was not necessarily the optimal time point for the study. Finally, selection bias is inevitable in this single-center retrospective study.

## Conclusion

This study demonstrates the significant impact of AISI on mortality in SA-AKI. AISI possesses an important predictive value in the SA-AKI prognosis, which serves as an efficient tool for prognostic evaluation in clinical practice and offers a theoretical basis for further optimizing the SA-AKI management strategy. The mortality in SA-AKI rises with the elevation of AISI when AISI > 450.117 × 10^18^/L. Quantitative stratification of AISI at admission may contribute to early detection and treatment of SA-AKI with a poor prognosis, but extensive prospective studies are still required to validate its clinical use.

## Supplementary Information

Below is the link to the electronic supplementary material.

Supplementary Material 1

## Data Availability

The data generated in this paper are available from the MIMIC-IV database (https://mimic.physionet.org).

## References

[1] Vincent, J. L. et al. Assessment of the worldwide burden of critical illness: the intensive care over nations (ICON) audit. *Lancet Respir Med.* **2**, 380–386. 10.1016/s2213-2600(14)70061-x (2014). 24740011 10.1016/S2213-2600(14)70061-X

[2] Li, Y. M. et al. Sepsis and acute kidney injury. *Natl. Med. J. China*. **101**, 1210–1213. 10.3760/cma.j.cn112137-20201201-03232 (2021). 10.3760/cma.j.cn112137-20201201-03232 34865390

[3] Bagshaw, S. M., George, C. & Bellomo, R. Early acute kidney injury and sepsis: a multicentre evaluation. *Crit. Care*. **12**, R47. 10.1186/cc6863 (2008). 18402655 10.1186/cc6863 PMC2447598

[4] Zarbock, A. et al. Sepsis-associated acute kidney injury: consensus report of the 28th acute disease quality initiative workgroup. *Nat. Rev. Nephrol.* **19**, 401–417. 10.1038/s41581-023-00683-3 (2023). 36823168 10.1038/s41581-023-00683-3

[5] Bai, X., Chen, T. & Lie, C. Research progress of Sepsis-Associated acute kidney injury. *Adv. Clin. Med.* **14**, 820–828. 10.12677/acm.2024.141115 (2024).

[6] Zinellu, A. et al. The aggregate index of systemic inflammation (AISI): A novel prognostic biomarker in idiopathic pulmonary fibrosis. *J. Clin. Med.* **10**10.3390/jcm10184134 (2021). 10.3390/jcm10184134 PMC8466198 34575245

[7] Ercan, Z. et al. The aggregate index of systemic inflammation May predict mortality in COVID-19 patients with chronic renal failure. *Eur. Rev. Med. Pharmacol. Sci.* **27**, 3747–3752. 10.26355/eurrev_202304_32173 (2023). 37140323 10.26355/eurrev_202304_32173

[8] Zinellu, A., Paliogiannis, P. & Mangoni, A. A. Aggregate index of systemic inflammation (AISI), disease severity, and mortality in COVID-19: A systematic review and Meta-Analysis. *J. Clin. Med.* **12**10.3390/jcm12144584 (2023). 10.3390/jcm12144584 PMC10381001 37510699

[9] Hosseninia, S., Ghobadi, H., Garjani, K., Hosseini, S. A. H. & Aslani, M. R. Aggregate index of systemic inflammation (AISI) in admission as a reliable predictor of mortality in COPD patients with COVID-19. *BMC Pulm Med.* **23**, 107. 10.1186/s12890-023-02397-5 (2023). 37003999 10.1186/s12890-023-02397-5 PMC10063934

[10] Ghobadi, H. et al. Role of leukocytes and systemic inflammation indexes (NLR, PLR, MLP, dNLR, NLPR, AISI, SIR-I, and SII) on admission predicts in-hospital mortality in non-elderly and elderly COVID-19 patients. *Front Med (Lausanne)* 9, 916453,10.3389/fmed.2022.916453 (2022). 10.3389/fmed.2022.916453 PMC9434555 36059829

[11] Xiu, J. et al. The aggregate index of systemic inflammation (AISI): a novel predictor for hypertension. *Front. Cardiovasc. Med.* **10**, 1163900. 10.3389/fcvm.2023.1163900 (2023). 37265570 10.3389/fcvm.2023.1163900 PMC10229810

[12] Jiang, Y. et al. Association between the aggregate index of systemic inflammation and clinical outcomes in patients with acute myocardial infarction: A retrospective study. *J. Inflamm. Res.* **17**, 7057–7067. 10.2147/jir.S481515 (2024). 39377046 10.2147/JIR.S481515 PMC11457786

[13] Göçmen, A. & Gesoglu Demir, T. The aggregate index of systemic inflammation as a predictor of mortality in stroke patients. *Cureus* **16**, e64007. 10.7759/cureus.64007 (2024). 39109115 10.7759/cureus.64007 PMC11301770

[14] Huang, Y. W., Zhang, Y., Li, Z. P. & Yin, X. S. Association between a four-parameter inflammatory index and all-cause mortality in critical ill patients with non-traumatic subarachnoid hemorrhage: a retrospective analysis of the MIMIC-IV database (2012–2019). *Front. Immunol.* **14**, 1235266. 10.3389/fimmu.2023.1235266 (2023). 37936706 10.3389/fimmu.2023.1235266 PMC10626529

[15] Yang, Y. et al. The systemic inflammation indexes predict all-cause mortality in peritoneal Dialysis patients. *Ren. Fail.* **45**, 2160348. 10.1080/0886022x.2022.2160348 (2023). 37417210 10.1080/0886022X.2022.2160348 PMC10332209

[16] Putzu, C. et al. Blood cell count indexes as predictors of outcomes in advanced non-small-cell lung cancer patients treated with nivolumab. *Cancer Immunol. Immunother*. **67**, 1349–1353. 10.1007/s00262-018-2182-4 (2018). 29947960 10.1007/s00262-018-2182-4 PMC11028046

[17] Guven, D. C. et al. The association between the Pan-Immune-Inflammation value and cancer prognosis: A systematic review and Meta-Analysis. *Cancers (Basel)*. 14. 10.3390/cancers14112675 (2022). 10.3390/cancers14112675 PMC9179577 35681656

[18] Wang, H. K. et al. Clinical usefulness of the lymphocyte-to-monocyte ratio and aggregate index of systemic inflammation in patients with esophageal cancer: a retrospective cohort study. *Cancer Cell. Int.* **23**, 13. 10.1186/s12935-023-02856-3 (2023). 36707809 10.1186/s12935-023-02856-3 PMC9881346

[19] Xie, W. et al. A novel nomogram combined the aggregate index of systemic inflammation and PIRADS score to predict the risk of clinically significant prostate cancer. *Biomed. Res. Int.* **2023** (9936087). 10.1155/2023/9936087 (2023). 10.1155/2023/9936087 PMC9851778 36685670

[20] Collins, G. S., Reitsma, J. B., Altman, D. G. & Moons, K. G. Transparent reporting of a multivariable prediction model for individual prognosis or diagnosis (TRIPOD): the TRIPOD statement. *Bmj* **350**, g7594. 10.1136/bmj.g7594 (2015). 25569120 10.1136/bmj.g7594

[21] Rhodes, A. et al. Surviving sepsis campaign: international guidelines for management of sepsis and septic shock: 2016. *Crit. Care Med.* **45**, 486–552. 10.1097/ccm.0000000000002255 (2017). 28098591 10.1097/CCM.0000000000002255

[22] Kellum, J. A. & Lameire, N. Diagnosis, evaluation, and management of acute kidney injury: a KDIGO summary (Part 1). *Crit. Care*. **17**, 204. 10.1186/cc11454 (2013). 23394211 10.1186/cc11454 PMC4057151

[23] Fucà, G. et al. The Pan-Immune-Inflammation value is a new prognostic biomarker in metastatic colorectal cancer: results from a pooled-analysis of the Valentino and TRIBE first-line trials. *Br. J. Cancer*. **123**, 403–409. 10.1038/s41416-020-0894-7 (2020). 32424148 10.1038/s41416-020-0894-7 PMC7403416

[24] van der Poll, T., Shankar-Hari, M. & Wiersinga, W. J. The immunology of sepsis. *Immunity* **54**, 2450–2464. 10.1016/j.immuni.2021.10.012 (2021). 34758337 10.1016/j.immuni.2021.10.012

[25] Zhang, Y., Peng, W. & Zheng, X. The prognostic value of the combined neutrophil-to-lymphocyte ratio (NLR) and neutrophil-to-platelet ratio (NPR) in sepsis. *Sci. Rep.* **14**, 15075. 10.1038/s41598-024-64469-8 (2024). 38956445 10.1038/s41598-024-64469-8 PMC11219835

[26] Xia, Y. et al. Prognostic value of neutrophil-to-monocyte/lymphocyte ratio for 28-day mortality in ICU sepsis patients: a retrospective cohort study. *Front. Med. (Lausanne)*. **11**, 1434922. 10.3389/fmed.2024.1434922 (2024). 39211344 10.3389/fmed.2024.1434922 PMC11358076

[27] Zheng, R., Shi, Y. Y., Pan, J. Y., Qian, S. Z. & Decrease in the platelet-to-lymphocyte ratio in days after admission for sepsis correlates with in-hospital mortality. *Shock* **59**, 553–559, doi:10.1097/shk.0000000000002087 (2023). 36802214 10.1097/SHK.0000000000002087

[28] Zheng, C. F. et al. Prognostic value of platelet-to-lymphocyte ratios among critically ill patients with acute kidney injury. *Crit. Care*. **21**, 238. 10.1186/s13054-017-1821-z (2017). 28882170 10.1186/s13054-017-1821-z PMC5590135

[29] Luo, X., Wan, D., Xia, R., Liao, R. & Su, B. Prognostic value of the baseline and early changes in Monocyte-to-Lymphocyte ratio for Short-Term mortality among critically ill patients with acute kidney injury. *J. Clin. Med.* **12**10.3390/jcm12237353 (2023). 10.3390/jcm12237353 PMC10707087 38068405

[30] Jiang, F. et al. Monocyte-to-lymphocyte ratio: a potential novel predictor for acute kidney injury in the intensive care unit. *Ren. Fail.* **44**, 1004–1011. 10.1080/0886022x.2022.2079521 (2022). 35672903 10.1080/0886022X.2022.2079521 PMC9186355

[31] Xiao, W. et al. Influence of the initial neutrophils to lymphocytes and platelets ratio on the incidence and severity of Sepsis-Associated acute kidney injury: A double robust Estimation based on a large public database. *Front. Immunol.* **13**, 925494. 10.3389/fimmu.2022.925494 (2022). 35903103 10.3389/fimmu.2022.925494 PMC9320191

[32] Piotrowski, I., Kulcenty, K. & Suchorska, W. Interplay between inflammation and cancer. *Rep. Pract. Oncol. Radiother*. **25**, 422–427. 10.1016/j.rpor.2020.04.004 (2020). 32372882 10.1016/j.rpor.2020.04.004 PMC7191124

[33] Nahrendorf, M. Myeloid cell contributions to cardiovascular health and disease. *Nat. Med.* **24**, 711–720. 10.1038/s41591-018-0064-0 (2018). 29867229 10.1038/s41591-018-0064-0 PMC7301893

[34] Cui, Y., Li, Y., Wang, Y. & Shang, K. Systemic immune-inflammation index predict sepsis mortality: a retrospective study. *Chin. J. Emerg. Med.* **14**, 820–828. 10.3760/cma.j.issn.1671-0282.2024.02.010 (2024).

[35] Bonventre, J. V. & Zuk, A. Ischemic acute renal failure: an inflammatory disease? *Kidney Int.* **66**, 480–485. 10.1111/j.1523-1755.2004.761_2.x (2004). 15253693 10.1111/j.1523-1755.2004.761_2.x

[36] Zhang, H. et al. Neutrophil, neutrophil extracellular traps and endothelial cell dysfunction in sepsis. *Clin. Transl Med.* **13**, e1170. 10.1002/ctm2.1170 (2023). 36629024 10.1002/ctm2.1170 PMC9832433

[37] Deng, B., Wang, S., Zhou, P. & Ding, F. New insights into immune cell diversity in acute kidney injury. *Cell. Mol. Immunol.* **20**, 680–682. 10.1038/s41423-023-01003-2 (2023). 36973486 10.1038/s41423-023-01003-2 PMC10229659

[38] Xiang, H., Xu, Z., Zhang, C. & Xiong, J. Macrophage-derived exosomes mediate glomerular endothelial cell dysfunction in sepsis-associated acute kidney injury. *Cell. Biosci.* **13**, 46. 10.1186/s13578-023-00990-z (2023). 36879272 10.1186/s13578-023-00990-z PMC9990300

[39] Chung, H., Lee, J. H., Jo, Y. H., Hwang, J. E. & Kim, J. Circulating Monocyte Counts and its Impact on Outcomes in Patients With Severe Sepsis Including Septic Shock. *Shock* 51, 423–429, 10.1097/shk.0000000000001193 (2019). 10.1097/SHK.0000000000001193 30286035

[40] Vardon-Bounes, F. et al. Platelets are critical key players in sepsis. *Int. J. Mol. Sci.* **20**10.3390/ijms20143494 (2019). 10.3390/ijms20143494 PMC6679237 31315248

[41] Camicia, G., Pozner, R. & de Larrañaga, G. Neutrophil extracellular traps in sepsis. *Shock* **42**, 286–294. 10.1097/shk.0000000000000221 (2014). 25004062 10.1097/SHK.0000000000000221

[42] Semple, J. W., Italiano, J. E. Jr. & Freedman, J. Platelets and the immune continuum. *Nat. Rev. Immunol.* **11**, 264–274. 10.1038/nri2956 (2011). 21436837 10.1038/nri2956

[43] Cox, D. Sepsis - it is all about the platelets. *Front. Immunol.* **14**, 1210219. 10.3389/fimmu.2023.1210219 (2023). 37350961 10.3389/fimmu.2023.1210219 PMC10282552

[44] Jansen, M. P. B., Florquin, S. & Roelofs, J. The role of platelets in acute kidney injury. *Nat. Rev. Nephrol.* **14**, 457–471. 10.1038/s41581-018-0015-5 (2018). 29760447 10.1038/s41581-018-0015-5

[45] Tan, D. & Peng, P. Immunopathology of neutrophil to lymphocyte ratio in sepsis and its diagnostic and prognostic value. *Adv. Clin. Med.* **13**, 1860–1867. 10.12677/acm.2023.132257 (2023).

[46] Ammer-Herrmenau, C. et al. Sepsis induces long-lasting impairments in CD4 + T-cell responses despite rapid numerical recovery of T-lymphocyte populations. *PLoS One*. **14**, e0211716. 10.1371/journal.pone.0211716 (2019). 30730978 10.1371/journal.pone.0211716 PMC6366777

[47] Le Tulzo, Y. et al. Early Circulating lymphocyte apoptosis in human septic shock is associated with poor outcome. *Shock* **18**, 487–494. 10.1097/00024382-200212000-00001 (2002). 12462554 10.1097/00024382-200212000-00001

[48] Bian, X. & Zhang, L. Research progress on predicting sepsis related acute kidney injury using multiple inflammatory markers. *Adv. Clin. Med.* **13**, 19162–19167. 10.12677/acm.2023.13122696 (2023).

