# Supplementary material for: The aggregate index of systemic inflammation is positively correlated with the risk of all-cause mortality in sepsis-associated acute kidney injury
Source: Sci Rep. 2025 Aug 15;15:29962. doi: 10.1038/s41598-025-16081-7 (PMC12356892; doi:10.1038/s41598-025-16081-7)
Supplement: Supplementary file 1 — Supplementary Material 1 [file 41598_2025_16081_MOESM1_ESM.docx]

**Table S1. Sensitivity analysis of COX regression**

|  | **AISI (10^18^/L)** | **Model 1** | | **Model 2** | | **Model 3** | |
| --- | --- | --- | --- | --- | --- | --- | --- |
|  |  | **HR(95%CI)** | **P value** | **HR(95%CI)** | **P value** | **HR(95%CI)** | **P value** |
| 30d | <735.405 | — | — | — | — | — | — |
|  | ≥735.405 | 1.371(1.251-1.502) | <0.001 | 1.303(1.189-1.429) | <0.001 | 1.174(1.069-1.290) | <0.001 |
| 90d | <735.405 | — | — | — | — | — | — |
|  | ≥735.405 | 1.409 (1.299-1. 528) | <0.001 | 1.335(1.238 -1.458) | <0.001 | 1.229(1.130-1.336) | <0.001 |
| 180d | <735.405 | — | — | — | — | — | — |
|  | ≥735.405 | 1.432(1.326-1.545) | <0.001 | 1.372(1.270-1.481) | <0.001 | 1.259(1.164-1.362) | <0.001 |
| 1year | <735.405 | — | — | — | — | — | — |
|  | ≥735.405 | 1.414(1.316-1.518) | <0.001 | 1.362(1.268-1.464) | <0.001 | 1.264(1.175-1.360) | <0.001 |
|  |  |  |  |  |  |  |  |
| Model 1: unadjusted | | | | | | | |
| Model 2: adjusted for gender, age, and race | | | | | | | |
| Model 3: adjusted for gender, age, race, BMI, temperature mean, heart rate mean,resp rate mean, SP, DP, mBP mean,SPO2 mean, bilirubin total, creatinine, ALT, AST, glucose mean, lactate, PT, PTT,GCS,SAPS II, Type 2 diabetes, CAHD, COPD, cirrhosis,congestive heart failure, cerebrovascular disease, peptic ulcer disease, antibiotic, norepinephrine, ventilation status, and CRRT  AISI, aggregate index of systemic inflammation; BMI, body mass index; SP, systolic pressure; DP, diastolic pressure; mBP, mean blood pressure; SPO2, percutaneous oxygen saturation; ALT, alanine aminotransferase;AST, Aspartate aminotransferase; PT, Prothrombin time; PTT, partial thromboplastin time; GCS, Glasgow Coma Scale; SAPS II, simplifed acute physiology score II; CRRT, continuous renal replacement therapy; CAHD, coronary atherosclerotic heart disease; COPD, chronic obstructive pulmonary disease; HR, Hazard Ratio; CI, Confidence Interval | | | | | | | |
